# Self-reported chronic diseases and health status and health service utilization - Results from a community health survey in Singapore

**DOI:** 10.1186/1475-9276-11-44

**Published:** 2012-08-16

**Authors:** Pradeep Paul George, Bee Hoon Heng, Joseph Antonio De Castro Molina, Lai Yin Wong, Ng Charis Wei Lin, Jason Tian Seng Cheah

**Affiliations:** 1 Health Services and Outcomes Research (HSOR), National Health Group, , Singapore; 2 Agency for Integrated Care, , Singapore

## Abstract

**Objective:**

To report the extent of self-reported chronic diseases, self-rated health status (SRH) and healthcare utilization among residents in 1-2 room Housing Development Board (HDB) apartments in Toa Payoh.

**Materials & methods:**

The study population included a convenience sample of residents from 931 housing development board (HDB) units residing in 1-2 room apartments in Toa Payoh. Convenience sampling was used since logistics precluded random selection. Trained research assistants carried out the survey. Results were presented as descriptive summary.

**Results:**

Respondents were significantly older, 48.3% reported having one or more chronic diseases, 32% have hypertension, 16.8% have diabetes, and 7.6% have asthma. Median SRH score was seven. Hospital inpatient utilization rate were highest among Indian ethnic group, unemployed, no income, high self-rated health (SRH) score, and respondents with COPD, renal failure and heart disease. Outpatient utilization rate was significantly higher among older respondents, females, and those with high SRH scores (7-10).

**Conclusions:**

The findings confirming that residents living in 1-2 room HDB apartments are significantly older, with higher rates of chronic diseases, health care utilization than national average, will aid in healthcare planning to address their needs.

## Introduction

Self-rated health and utilization of healthcare services are important determinants of health, and have particular relevance for public health [1]. Health service utilization is a complex behavioral phenomenon, related to the availability, quality, cost and comprehensiveness of services as well as socio-cultural structure, health beliefs and personal characteristics of the users [2]. There is reason to believe that people reporting better health status are less frequent users of healthcare services [3]. Understanding how self-perceived health status influences healthcare utilization may help estimate future demand for physicians, and healthcare planning. In Singapore, the rapid ageing of the baby-boomer generation poses a serious challenge to healthcare providers and policy makers [4]. Ageing will result in an increase in chronic disease morbidity, reduced levels of functioning, and increased disability, leading to increased medical care and healthcare expenditures. Studies show that the people with low income/socio-economic status (SES) have more health related problems than people with high SES [3-8]. This group exhibit greatest need for healthcare and are the most frequent users of healthcare services [2,9].

Healthcare utilization information for government outpatient facilities and hospitals are readily available from statistics of clinic attendances and inpatient discharges. However, current information on people’s healthcare preference, health seeking behavior, functional assessment and perception about their health status are either not available or not up to date [5,6]. Community health assessment helps us to understand the health problems and priorities of a population.

The National Healthcare Group (NHG) embarked on a community health assessment, aimed to determine the prevalence of self-reported chronic diseases and risk factors, functional status, lifestyle behavior, willingness to participate in health programs, and utilization of health services. It provides baseline information to support program planning for groups at greater risk of chronic diseases, who may potentially have limited access to healthcare, and to identify opportunities for disease prevention and health promotion. The aim of this study was to describe the self-reported chronic diseases, health status and health service utilization of residents living in 1–2 room Housing Development Board (HDB) apartments in Toa Payoh.

## Methods

The study population included a convenience sample of residents from 931 housing development board (HDB) units residing in 1–2 room apartments in Toa Payoh. Convenience sampling was used since logistics precluded random selection. These 1–2 room HDB units are heavily subsidized by the government’s Housing and Development Board to the poor and needy citizen families with no housing options and with a gross household monthly income of below S$2,000 (~ USD 1589) for purchase, or below S$1,500 (~ USD 1191) for rent [10]. Of the 1- and 2-room resident population in Singapore, 9.4% of them reside in Toa Payoh [11,12]. The house-to-house survey was conducted between June and October 2009 in two stages: (a) a household survey enumerated all persons in the households to derive eligible respondents, telephone contact, language medium for quality audits and future contacts. It provided an estimate of the total study population, which served as the denominator for indicators captured in the survey; and (b) an individual survey to elicit the information to address the specific aims of the project. Housing units where none of the residents could be contacted after three visits on three separate days were excluded. Respondents were eligible for the individual survey if they were household members and were Singapore citizens or Permanent Residents. A household member was defined as one who considered the house as his/her main dwelling place, slept in the house at least 4 nights per week, and/or was in the house for most part of the day (≥8 hours) and/or shared the same eating arrangements for most part of the week (≥5 times a week) in the preceding month. Foreigners, work permit and employment pass holders staying at the selected household were excluded. Except for those with asthma, respondents should have been at least 18 years old. Patients with asthma who were at least 7 years old were interviewed. Proxy respondents who were sufficiently familiar with circumstances of the person he/she was representing, e.g. parent, child, main caregiver, other close relative, were allowed for cognitively and mentally challenged household members, and children with asthma below 7 years of age. Eligible respondents who were not contactable after three attempts on three separate days were excluded. After obtaining the verbal consent, interviewers provided the respondents with a survey overview and expected duration of completion. Individual’s responses were recorded in the study questionnaire (Additional File: 1).

Eligible respondents were asked if they had been diagnosed by a western doctor for eight major chronic diseases such as hypertension, diabetes, hypercholesterolemia, heart disease, stroke, renal failure, asthma, COPD and other medical conditions; their actual health service utilization and preference of provider. Respondents were asked to rate their health status on a likert scale from 1–10, with a score of one indicating poor health and a score of ten indicating best possible health. The developed questionnaire (Additional File: 1) was subjected to cognitive testing, field-tested, translated and back translated from English to Mandarin and Malay. Face-to-face interviews were conducted in English, Mandarin, Malay or Chinese dialects by a team of trained research assistants. The completed survey forms were subjected to quality audit and subsequently entered into an MS Access database with inbuilt validity checks to reduce data entry errors. Ethics approval for the survey was obtained from the Domain Specific Review Board of the National Healthcare Group.

### Statistical analysis

Age, work status, income were grouped into broader categories for analysis. Continuous variables were expressed as mean ± SD or median (intra-quartile range) and categorical variables were reported as percentages. Associations of self-reported chronic diseases, health status and healthcare utilization with socio-demographic variables and other risk factors were assessed by Pearson chi-square tests or *t*-test where appropriate. Multivariate logistic regression (enter method) analysis was carried out to determine factors significantly associated with self-rated health, in-patient and out-patient utilization, variables included in the equation were age, gender, ethnicity, education, work status, monthly income, self-rated health and chronic diseases. To avoid over-fitting the model, only variables found significant on univariate analysis were included in the final regression model. All statistical analysis were performed using Predictive Analytics SoftWare (PASW) Statistics Version 18. All tests were conducted at the 5% level of significance. The percentages and the Odds ratio (OR) were reported with their 95% Confidence Intervals (CI) where applicable.

## Results

### Demographic and other characteristics

The overall survey response rate was 50.0%; household and individual response rate were 62.6% and 79.9% respectively. Of the total 1,145 residents staying in 558 households, 991 residents were eligible for the survey, of whom 778 were successfully interviewed (Table 1). Survey respondents were older, had higher percentage of Malays, higher percentage with no formal education and higher percentage of unemployed persons and higher percentage with income less than $500 than the national average (Table 2). Average age of the respondents was 56.2 ± 18.4 years. 63.7% of the respondents were Chinese, 22.6% were Malays and 9.8% were Indians. 48.1% of the respondents had no formal education, 29.7% had primary education and 22.2% had tertiary education. 67% of the respondents had monthly personal income of < S$500 (Singapore dollars) and 22.9% earned between S$500 – 999 and 10.1% earned ≥ S$1000.

**Table 1:** Response rate

**Total no. of housing units in 4 blocks**	**931**
**Household survey:**	
- vacant (excluded)	39
No. of units visited	892
- no answer after 3 visits	168
- refused	166
No. of units agreed to participate (a)	558
**Household response rate**	**558/(892) = 62.6%**
**Individual survey:**	
Total no. of residents in (a)	1,145
- eligible	974
- refused	109
- not contactable after 3 attempts (excluded)	87
No. of residents successfully surveyed	778
**Individual response rate**	**778/974 = 79.9%**
**Overall response rate**	**62.6% x 79.9% = 50.0%**

**Table 2:** Comparison of demographic characteristics of survey respondents with Singapore general population

**Characteristics**	**Categories**	**Survey respondents**	**%**	**95% CI**^**┼**^	Singapore population*%
Age	<25	53	6.8	5.3 - 8.9	31.4
	25 - 44	132	17.1	14.5 - 19.9	32.2
	45 - 64	300	38.8	35.4 - 42.4	27.6
	≥65	288	37.3	33.9 - 40.9	8.8
Gender	Male	390	50.1	45.1 - 55.1	49.5
	Female	388	49.9	44.9 - 54.9	50.5
Ethnic group	Chinese	496	63.8	59.8 - 67.8	74.7
	Malay	176	22.6	16.6 - 28.6	13.6
	Indian	76	9.8	2.8 - 16.8	8.9
	Others	30	3.9	0.0 - 10.9	2.8
Highest educational level attained	No formal education	374	48.1	43.1 - 53.1	16.4
	Primary	231	29.7	23.7 - 35.7	22.0
	Secondary and higher	173	22.2	16.2 - 28.2	61.6
Work status	Employed (Full time/Part-time)	335	43.1	38.1 - 48.1	96.0
	Unemployed	157	20.2	14.2 - 26.2	4.0
Personal monthly income	< S$500	521	67	63.0 - 71.0	3.7
	S$500 - S$999	178	22.9	16.9 - 28.9	9.5
	≥ S$ 1000	79	10.1	3.1 - 17.1	86.8

*Sources: Yearbook of Statistics 2009 & General Household Survey 2005 accessed from http://www.singstat.gov.sg/pubn/popn/population2009.pdf,

http://www.singstat.gov.sg/pubn/popn/ghsr1.html.

^┼^CI: Confidence Interval.

### Self-reported chronic diseases

The prevalence of self-reported eight major chronic diseases was 48.3% (n = 376, 95% Confidence Interval (CI): 44.8 – 51.9%). Twenty-two percent reported having one chronic disease (n = 169), 14% reported having two diseases (n = 109), 8% reported having three diseases (n = 64), and 4% reported having been diagnosed with four or more diseases (n = 34). Self-reported prevalence of hypertension, diabetes, heart disease and asthma were 32%, 16.8%, 21.1% and 7.6%, respectively. Other chronic diseases reported include mental problems (2.4%), musculoskeletal problems (2.1%), skin problems (1.2%), eye problems (0.9%), gastrointestinal problems (0.8%) and cancer (0.5%). The rate of self-reported chronic diseases increased progressively with age, and was higher among females, Chinese ethnic group, retirees, unemployed, respondents with monthly income < $500 and those whose self-rated health (SRH) score (1–6) (Table 3). Self-reported chronic diseases were classified as one or more chronic disease and no chronic disease, multivariate logistic regression was performed to identify the significant predictors. Presence of one or more chronic disease significantly predicted the resident’s self-rated health, inpatient and outpatient utilization. Residents with one or more chronic disease were 3 times more likely to report poor self-rated health (Odds Ratio (OR): 3.05, 95% (CI): 2.09 – 4.44), 2 times more likely to report inpatient utilization (OR: 2.25, 95% CI: 1.25 – 4.03) and 36 times more likely to have outpatient utilization (OR: 36.2, 95% CI: 17.31 – 17.55) than residents with no chronic disease (Table 4).

**Table 3:** **Self-reported chronic diseases**^**# **^**and health service utilization by various characteristics (n = 778)**

**Characteristics**	**Categories**	**n**	**Row%**			
			**Self-reported chronic disease**	**Outpatient utilization rate**	**Inpatient utilization rate**	**SRH score 1-6**
Age-group*	<25	53	26.4	83.0	11.3	13.2
	25 - 44	132	34.8	78.0	8.3	29.5
	45 - 64	300	54.8	78.7	10.0	33.2
	≥65	288	80.6	91.0	11.1	41.3
Gender	Males	390	53.1	79.0	10.8	32.3
	Females	388	65.5	88.1	9.8	36.6
Ethnicity	Chinese	496	62.7	83.5	9.5	38.1
	Malay	176	51.1	85.2	9.7	24.4
	Indian	76	60.5	85.5	18.4	34.2
	Others	30	46.7	70.0	6.7	33.3
Highest educational level attained	No formal education	374	70.6	85.0	12.8	40.1
	Primary	231	55.0	80.1	6.5	32.9
	Secondary education and above	173	40.5	84.9	9.8	24.3
Work status	Working full-time or part-time	335	43.6	76.7	8.4	23.6
	Unemployed	157	69.4	85.4	16.6	41.2
	Retiree	134	82.8	91.0	9.0	39.6
	Housewife	129	72.1	89.9	10.9	44.2
	Student or national serviceman	23	8.3	87.5	0.0	8.7
Personal Monthly Income^╣^	<S$500	421	71.7	88.5	12.4	44.2
	S$500 - 999	278	47.5	76.9	8.6	24.1
	≥S$1,000	79	34.2	79.7	5.1	19.0
Self-rated health status	Score 1-6	268	77.9	91.8	13.8	-
	Score 7-10	510	49.4	79.2	8.4	-
Chronic diseases	Presence of one or more chronic disease	461	-	98.0	13.2	45.3
	No chronic disease	317	-	62.4	6.0	18.6

^#^ refers to hypertension, diabetes, hypercholesterolemia, heart disease, stroke, renal failure, asthma, COPD, cancer and other mental, musculoskeletal, skin, eye, gastrointestinal problems.

* Age not available for five respondents, income not available for 3 respondents.

SRH: Self-reported health; score of 1 indicates poor health and a score of 10 indicates best possible health. Scores 1–6 indicate fair to poor self-rated health status.

^╣^Income in Singapore dollars.

**Table 4:** Determinants of Self-reported health and health service utilization by various characteristics (n = 778)

**Characteristics**	**Categories**	**Self- rated health (1–6), OR**	**95% CI for OR**		**Inpatient utilization, OR**	**95% CI for OR**		**Outpatient utilization, OR**	**95% CI for OR**	
			**Upper**	**Lower**		**Upper**	**Lower**		**Upper**	**Lower**
Age-group*	<25	1			1			1		
	25 - 44	2.27	0.84	6.14	0.51	0.16	1.63	1.66	0.57	4.87
	45 - 64	1.78	0.66	4.77	0.49	0.17	1.49	1.31	0.45	3.83
	≥65	1.38	0.49	3.92	0.44	0.13	1.46	2.09	0.59	7.35
Gender	Males	1			1			1		
	Females	0.94	0.65	1.35	0.68	0.39	1.17	1.55	0.92	2.61
Ethnicity	Chinese	1			1			1		
	Indian	0.92	0.52	1.61	2.35	1.15	4.77	1.02	0.45	2.35
	Others	0.98	0.42	2.29	0.70	0.16	3.19	0.42	0.15	1.17
	Malay	0.57	0.37	0.88	1.23	0.64	2.35	1.49	0.84	2.64
Highest educational level attained	No formal education	1.16	0.70	1.92	1.06	0.52	2.16	0.41	0.20	0.81
	Primary	1.12	0.67	1.86	0.45	0.20	1.02	0.52	0.27	1.00
	≥ Secondary education	1			1			1		
Work status	Working full-time or part-time	1			1			1		
	Retiree	0.68	0.23	1.96	0.23	0.03	1.75	0.71	0.17	3.00
	Housewife	0.89	0.30	2.66	0.32	0.04	2.36	0.86	0.21	3.52
	Student or national serviceman	0.37	0.06	2.41	0.00	-	-	4.89	0.75	31.83
	Unemployed	1.05	0.37	2.97	0.39	0.06	2.78	0.84	0.24	2.99
Personal Monthly Income^╣^	<S$500	3.11	0.94	10.27	8.15	0.90	73.78	0.76	0.18	3.17
	S$500 - 999	1.25	0.63	2.49	2.09	0.65	6.65	0.66	0.32	1.39
	≥S$1,000	1			1			1		
Chronic diseases	Presence of one or more chronic disease	3.05	2.09	4.44	2.25	1.25	4.03	36.17	17.31	75.55
	No chronic disease	1			1			1		

* Age not available for five respondents, income not available for 3 respondents.

OR: Odds ratio, CI: Confidence Interval, ^╣^Income in Singapore dollars.

SRH: Self-reported health; score of 1 indicates poor health and a score of 10 indicates best possible health. Scores 1–6 indicate fair to poor self-rated health status.

### Healthcare utilization

#### Inpatient utilization

Overall, 10.6% of the survey respondents (n = 83, 95% CI: 8.6 – 13.1%) were hospitalized in the preceding 12 months. Respondents with eight listed chronic diseases had significantly higher hospital utilization (12.5%) in comparison to respondents with no known medical diseases (6%) (p = 0.003). Similarly respondents who were younger (< 25 years), belonging to Indian ethnic group, those with no formal education, those who were unemployed, those with monthly income < S$500, those who self-rated their health score 1–6, and patients with COPD, renal failure and heart disease had higher inpatient utilization (Table 3). Independent predictors of inpatient utilization were ethnic group and presence of chronic diseases. Patients from Indian ethnicity had higher odds for inpatient utilization in comparison with their Chinese counterparts (OR: 2.35, 95% CI: 1.15 – 4.77); similarly patients with chronic diseases had significantly higher inpatient utilization than those without chronic diseases (OR: 2.25, 95% CI: 1.25 – 4.03) (Table 4).

#### Outpatient utilization

Eighty four percent of the respondents had visited an outpatient clinic in the preceding 12 months (n = 654, 95% CI: 81.3 – 86.5%), with a total of 4,225 outpatient visits made by 654 respondents, an average of 6.5 outpatient visits per person per year. Older respondents (≥ 65 years), females, Indians, respondents with no formal education, Retirees, respondents with monthly income < $500 and those with self- rated health (SRH) scores (1–6) had high outpatient utilization (Table 3). Presence of any chronic disease was an independent predictor of outpatient utilization; patients with chronic disease had higher odds for outpatient utilization when compared to those with no chronic disease (OR: 36.17, 95% CI: 17.31 – 75.55) (Table 4). Among respondents with 8 chronic diseases seeking treatment at the polyclinics, the rate of utilizing polyclinics increased progressively with age; and a higher proportion of younger respondents (<45 years) with 8 chronic conditions sought treatment at the general practitioner (GP) (Table 5).

**Table 5:** Age-specific utilization rate (%) of primary care services in preceding year

**Age-group**	**Utilization rate (%)**					
	**Any chronic disease**			**No chronic disease**		
	n*	**Government polyclinic**	**General practitioner**	n*	**Government polyclinic**	**General practitioner**
≤ 25	11	27.3	36.4	39	33.3	41.0
25 - 44	32	28.1	65.6	86	32.6	33.7
45 - 64	122	53.3	23.8	136	35.3	16.2
≥ 65	208	63.5	25.9	56	26.8	21.4
Total	373			317		

* Patients could have utilized more than one or more primary care service, hence the numbers would not add up to total (n = 778).

Polyclinic was the popular choice for the treatment of chronic diseases for 55% of the respondents, main reasons being cheap and accessible. For respondents who preferred to consult at specialist outpatient clinic, reasons for preference were accessibility, loyalty/familiarity, doctor-related factors and their perception that their medical conditions could be better managed. Similarly, the decisions to seek treatment at GP clinics were for accessibility, doctor-related factors and service quality (Table 6).

**Table 6:** Reason for choice of outpatient healthcare provider*

**Reasons for choice**	**% respondents who cited their preference for care providers**		
	**Government polyclinics**	**Specialist outpatient clinics in government hospitals**	**Private general practitioners**
	**n = 342**	**n = 120**	**n = 164**
Cheap	72.5	25.8	23.8
Accessible	66.1	37.5	59.8
Loyalty/familiarity	13.2	20.0	25.6
Service quality	10.8	5.8	21.3
Disease can be better managed	9.6	61.7	18.9
Doctor-related factors	8.8	30.0	23.8
Efficient	5.0	9.2	17.7
Recommended by others	3.8	10.0	5.5
More advanced technology	2.0	15.0	2.4
Good reputation	1.8	6.7	3.0

* Respondents may cite more than one reason for their choice and could have visited more than one outpatient healthcare provider.

### Self-rated health status

The median SRH score was seven (interquartile range, 6–8). Thirty-six percent of women and thirty two percent of men reported below median SRH scores. There was a discernible disparity among those with 8 chronic diseases (47%) and those with no known medical conditions (19%), respectively, reporting below median scores (p = 0.0001). Approximately 2 in 5 of respondents aged 65+ years, those with no formal education, either unemployed, retired or were housewives, were without income or belonging to Chinese ethnic group had below median SRH scores (Table 3). Presence of any chronic disease was an independent predictor of poor self-rated health status (SRH score 1–6), respondents with any chronic disease had higher odds of reporting poor self-rated health when compared to those without chronic disease (OR: 3.05, 95% CI: 2.09 – 4.44) (Table 4).

## Discussion

This study shows the self-reported rates of hypertension, diabetes, high blood cholesterol and asthma and inpatient utilization were higher than the national rates [13]. A third of respondents reported fair to poor SRH, similar to another study among 1–2 room residents [14] but higher than another report from Singapore [15]. The proportion of respondents with fair or poor health were higher among older age groups, among respondents who were not gainfully employed and among those with multiple chronic diseases, similar to that reported in other studies [13,14,16,17]. In a survey by Fong et al. [11] in the vicinity of the present survey, inpatient utilization was higher for females and those aged 60+ years. Our results showed that inpatient and outpatient utilization to be associated with presence of self-reported chronic conditions. The median age of survey respondent was 14 years older than that of the Singapore population, and not surprisingly a higher prevalence of chronic diseases. With ageing, this phenomenon will be further compounded. In Canada, an estimate of the impact of population ageing shows that the prevalence of chronic diseases would increase by more than 25% within the next quarter century and that this will result in health resource requirements growing more rapidly than the population, twice as rapidly in the case of hospital stays [18]. Another study found that levels of socio-economic status-based health inequality in a cohort progressively increase as the cohort ages [19].

Government polyclinics were the preferred as the primary care provider (55%), higher than 23.6% in the general population. The reverse holds true for private GPs with 75.9% of the general population preferring to seek treatment at GPs compared to 16% among respondents. This is expected. With the significantly lower median household income of S$1,090 among persons living in 1-2-room apartments compared to the national S$4,950, [17] makes the heavily subsidized government polyclinics a more affordable option for these residents. The study also showed that a higher proportion of persons with chronic disease gravitate to the polyclinics, similar to the 2007 National Health Surveillance Survey. The implication is with the significantly older residents in 1–2 room apartments that are associated with higher prevalence of chronic diseases; demand for government polyclinic services for this segment of population will further compound the national demand as the population ages.

### Strengths and limitations

The study highlights the healthcare disparities of a country with rapidly aging population. The study has several limitations that need to be acknowledged. This was a cross-sectional descriptive study and hence unable to show the temporal relationship between self-reported prevalence, self-rated health and healthcare utilization. The findings of this community health assessment had limited generalizability to the population from 1- and 2-room apartments beyond the Toa Payoh district within which the data were collected. The design of the survey is subject to recall bias. These limitations notwithstanding, this study has uncovered or confirmed significant findings useful for health planners in countries with rapidly aging populations.

## Conclusion

Residents in 1–2 room apartments are significantly older, a lower proportion being gainfully employed earning a significantly lower income, a higher proportion had chronic diseases and utilizes government polyclinics, and lower self-rated health status. The disparity in health status among persons with low and high income being greatest among the older people needs to be addressed.

## Competing interests

The authors declare that they have no competing interests.

## Authors’ contributions

PPG, HBH, JADM, WLY, NGCWL and CJTS assisted with the conceptualization and the actual design of the study. PPG carried out the statistical analysis and drafted the manuscript along with HBH and JADM. WLY, NGCWL and CJTS assisted with drafting the manuscript. All authors read and approved the final manuscript.

## Supplementary Material

Additional file 1 Survey of knowledge, self-empowerment and health-service utilization for chronic diseases. Click here for file

## References

[B1] Chakraborty N Islam MA Chowdhury RI Bari W Akhter HH Determinants of the use of maternal health services in rural Bangladesh Health Promot Int 2003 18 4 327 337 10.1093/heapro/dag414 14695364

[B2] Solome B Sarah W Sandro G Stefan P George P Community perceptions and factors influencing utilization of health services in Uganda 2009 10.1186/1475-9276-8-25 PMC2717964 19602244

[B3] Syed HR Dalgard OS Hussain A Dalen I Claussen B Ahlberg NL Inequalities in health: a comparative study between ethnic Norwegians and Pakistanis in Oslo, Norway Int J Equity Health 2006 5 1 7 10.1186/1475-9276-5-7 16808838 PMC1553452

[B4] Smith GD Blane D Bartley M Explanations for socio-economic differentials in mortality Eur J Publ Health 1994 4 2 131 144 10.1093/eurpub/4.2.131

[B5] Dalstra JAA Kunst AE Borrell C Breeze E Cambois E Costa G Geurts JJM Lahelma E Van Oyen H Rasmussen NK Socioeconomic differences in the prevalence of common chronic diseases: an overview of eight European countries Int J Epidemiol 2005 34 2 316 326 10.1093/ije/dyh386 15737978

[B6] Demakakos P Nazroo J Breeze E Marmot M Socioeconomic status and health: the role of subjective social status Soc Sci Med 2008 67 2 330 340 10.1016/j.socscimed.2008.03.038 18440111 PMC2547480

[B7] Siegrist J Marmot M Health inequalities and the psychosocial environment–two scientific challenges Soc Sci Med 2004 58 8 1463 1473 10.1016/S0277-9536(03)00349-6 14759690

[B8] Mackenbach JP Kunst AE Cavelaars AEJM Groenhof F Geurts JJM Socioeconomic inequalities in morbidity and mortality in western Europe* Lancet 1997 349 9066 1655 1659 10.1016/S0140-6736(96)07226-1 9186383

[B9] Wong M Chau PH Goggins W Woo J A geographical study of health services utilization among the elderly in Hong Kong: from spatial variations to health care implications Health Serv Insights 2009 2 1 12

[B10] Housing & Development Board. HDB InfoWEBwww.hdb.gov.sg

[B11] Fong NP Phua KH Utilization and expenditure on medical services in a local community Singapore Med J 1985 26 2 131 138 3929389

[B12] Ministry of Trade & Industry Singapore Census of Population 2000: Geographic distribution and travel: Singapore Department of Statistics 2001 http://www.singstat.gov.sg/pubn/popn/cop2000admin.pdf

[B13] National Health Surveillance Survey 2007 http://www.moh.gov.sg/mohcorp/publicationsreports.aspx?id=22520

[B14] Ramkumar A Quah JLS Yeo TWLSH Nieh CC Shankar A Wong TY Self-rated health, associated factors and diseases: a community-based cross-sectional study of Singaporean adults aged 40 years and above Ann Acad Med Singapore 2009 38 7 606 19652852

[B15] Lim WY Ma S Heng D Bhalla V Chew S Gender, ethnicity, health behaviour & self-rated health in Singapore BMC Publ Health 2007 7 1 184 10.1186/1471-2458-7-184 PMC1976324 17655774

[B16] Leslie K International comparison of key healthcare utilization trends MoH Information Paper 2004/04 www.moh.gov.sg/mohcorp/…/Information…/International_Comparison_of_Key_Healthcare

[B17] Department of Statistics, Singapore. Press releasehttp://www.singstat.gov.sg/news/news/op19022010.pdf

[B18] Denton FT Spencer BG Chronic health diseases: changing prevalence in an aging population and some implications for the delivery of healthcare serviceshttp://socserv.mcmaster.ca/sedap/p/sedap259.pdf 10.1017/S0714980809990390 20202262

[B19] Prus S Age, SES, and health: a population level analysis of health inequalities over the life coursehttp://socserv2.socsci.mcmaster.ca/~sedap/p/sedap181.pdf 10.1111/j.1467-9566.2007.00547.x 17381817

