# Supplementary material for: Self-reported chronic diseases and health status and health service utilization - Results from a community health survey in Singapore
Source: Int J Equity Health. 2012 Aug 16;11:44. doi: 10.1186/1475-9276-11-44 (PMC3490941; doi:10.1186/1475-9276-11-44)
Supplement: Additional file 1 — Survey of knowledge, self-empowerment and health-service utilization for chronic diseases. [file 1475-9276-11-44-S1.doc]

Reference No.:

-

Name of Respondent: ______________________________

Name of Proxy (if any): ______________________________

**SURVEY OF KNOWLEDGE, SELF-EMPOWERMENT AND HEALTH-SERVICE UTILIZATION FOR CHRONIC DISEASES**

| **A. Chronic Diseases** |
| --- |

INTERVIEWERS: Please complete all questions for each disease before moving on to the next

|  | | **Hypertension** | **Diabetes** | **Hypercho-lesterolemia** | **Heart Disease** | **Stroke** | **Renal Failure** | **Asthma** | **COPD** |
| --- | --- | --- | --- | --- | --- | --- | --- | --- | --- |
| A1 Have you been diagnosed by a  Western doctor to have any of  these conditions? | |  |  |  |  |  |  |  |  |
| **1** | Yes |
| **2** | No **[Go to Section B]** |
| **3** | Don’t know **[Go to A3]** |
| A2 When was it diagnosed? | |  |  |  |  |  |  |  |  |
| **1** | Within the past 12 months |
| **2** | Between 1 to 5 years ago |
| **3** | More than 5 years ago |
| A3 When did you have your last  check-up or test (excluding self-  monitoring) for your condition? | |  |  |  |  |  |  |  |  |
| **1** | ≤ 1 month |
| **2** | > 1 month to ≤ 3 months |
| **3** | > 3 months to ≤ 6 months |
| **4** | > 6 months to ≤ 1 year |
| **5** | > 1 year to ≤ 2 years |
| **6** | > 2 years |
| A4 Where are you being followed up  for your condition?  **(List all that apply)** | |  |  |  |  |  |  |  |  |
| **1** | Private GP (Family Clinic) |
| **2** | Polyclinic |
| **3** | SOC - Restructured Hospital **[Specify name in column]** |
| **4** | SOC - Private Hospital |
| **5** | Traditional medicine practitioner |
| **6** | Not seeking treatment |
| **7** | Others **[Specify in column]** |

|  | | **Hypertension** | **Diabetes** | **Hypercho-lesterolemia** | **Heart Disease** | **Stroke** | **Renal Failure** | **Asthma** | **COPD** |
| --- | --- | --- | --- | --- | --- | --- | --- | --- | --- |
| A5 How many times have you sought  treatment at the following in the  past 12 months? | |  |  |  |  |  |  |  |  |
| - | Private GP (Family Clinic) |  |  |  |  |  |  |  |  |
| - | Polyclinic |  |  |  |  |  |  |  |  |
| - | SOC - Restructured Hospital  **[Specify name in column]** |  |  |  |  |  |  |  |  |
| - | SOC - Private Hospital |  |  |  |  |  |  |  |  |
| - | Traditional medicine practitioner **[To answer A7 also]** |  |  |  |  |  |  |  |  |
| - | Emergency Department |  |  |  |  |  |  |  |  |
| A6 What are the reasons for  choosing the most frequently  visited healthcare provider (A5):  _________________________?  **(List all that apply)** | |  |  |  |  |  |  |  |  |
| **1** | Cheaper |
| **2** | More accessible |
| **3** | Condition can be better managed |
| **4** | Doctor-related factor |
| **5** | Service quality |
| **6** | More advanced technology |
| **7** | Reputation |
| **8** | Efficiency |
| **9** | Loyalty / familiarity |
| **10** | Recommendation |
| **11** | Others **[Specify in column]** |
| A7 What are the reasons for  choosing the traditional medicine  practitioner?  **(List all that apply)** | |  |  |  |  |  |  |  |  |
| **1** | Cheaper |
| **2** | More accessible |
| **3** | Condition can be better managed |
| **4** | Practitioner-related factor |
| **5** | Loyalty / familiarity |
| **6** | Western medical treatment not effective |  |
| **7** | Want a second opinion |
| **8** | Body modulation |
| **9** | Others **[Specify in column]** |
| A8 Were you prescribed any  Western medications for your  condition? | |  |  |  |  |  |  |  |  |
| **1** | Yes |
| **2** | No **[Go to A11]** |

|  | | **Hypertension** | **Diabetes** | **Hypercho-lesterolemia** | **Heart Disease** | **Stroke** | **Renal Failure** | **Asthma** | **COPD** |
| --- | --- | --- | --- | --- | --- | --- | --- | --- | --- |
| A9 Are you taking your Western  medications as instructed? | |  |  |  |  |  |  |  |  |
| **1** | Yes **[Go to A11]** |
| **2** | No |
| A10 Why have you not been taking  your Western medications as  instructed?  **(List all that apply)** | |  |  |  |  |  |  |  |  |
| **1** | Inconvenient /difficult to follow instructions |
| **2** | Expensive |
| **3** | Unable to go to pharmacy |
| **4** | Feeling better |
| **5** | Side effects |
| **6** | Forgot to take |
| **7** | Not effective |
| **8** | Others **[Specify in column]** |
| A11 What else are you currently doing  to manage your condition?  **(List all that apply)** | |  |  |  |  |  |  |  |  |
| **1** | Nothing |
| **2** | Exercise |
| **3** | Dietary control |
| **4** | Others **[Specify in column]** |
| A12 How many times were you  admitted to a hospital because of  the condition in the past 12  months?  **[Specify hospital name in**  **column]** | |  |  |  |  |  |  |  |  |
| **1** | Never |
| **2** | 1 time |
| **3** | 2 times |
| **4** | ≥ 3 times |
| A13 How do you usually pay for your  outpatient fees (consultation /  medication / lab tests) to the  healthcare provider?  **(List all that apply)** | |  |  |  |  |  |  |  |  |
| **1** | Out of own pocket |
| **2** | Company pays for it |
| **3** | Medisave |
| **4** | Insurance |
| **5** | Medifund |
| **6** | Paid by children / relatives |
| **7** | Other government scheme  **[Specify schemes in column]** |
| **8** | Other non-government scheme **[Specify schemes in column]** |
| **9** | Don’t know |

|  | | **Hypertension** | **Diabetes** | **Hypercho-lesterolemia** | **Heart Disease** | **Stroke** | **Renal Failure** | **Asthma** | **COPD** |
| --- | --- | --- | --- | --- | --- | --- | --- | --- | --- |
| A14 Who is your main caregiver  (assist in feeding / transferring /  toileting / dressing & undressing /  bathing)? | |  |  |  |  |  |  |  |  |
| **1** | Self **[Go to A16]** |
| **2** | Spouse |
| **3** | Children |
| **4** | Relatives |
| **5** | Friends |
| **6** | Maid |
| **7** | Others: _________________ |
| A15 Is your main caregiver staying  with you? | |  |  |  |  |  |  |  |  |
| **1** | Yes |
| **2** | No |
| A16 What kind(s) of assistance would  you like to have for your medical  condition?  **(List all that apply)** | |  |  |  |  |  |  |  |  |
| **1** | None |
| **2** | Social activities |
| **3** | Emotional support |
| **4** | Transportation |
| **5** | Financial assistance |
| **6** | Health programs (screening / talk) |
| **7** | Caregiver (feeding / transferring / toileting / dressing & undressing / bathing) |
| **8** | Infrastructural support (eg. hand rails / ramps) |
| **9** | Others **[Specify in column]** |
| A17 Aside from your usual healthcare  provider, do you want to obtain  information about your condition  from other sources? | |  |  |  |  |  |  |  |  |
| **1** | Yes |
| **2** | No **[End or Go to A19/22]** |
| A18 From which source would you  prefer to obtain extra information  about your disease? | |  | **[Go to A22]** | **[Go to Sect.C]** | **[Go to Sect.C]** | **[Go to Sect.C]** | **[Go to Sect.C]** | **[Go to Sect.C]** | **[Go to Sect.C]** |
| **1** | Relatives, friends, acquaintances |
| **2** | Media (eg. TV, radio, newspaper) |
| **3** | Books |
| **4** | Electronic sources (eg. Internet) |
| **5** | Talks, seminars, workshops |
| **6** | Others **[Specify in column]** |
| A19 Do you monitor your blood  pressure at home? | |  |  |  |  |  |  |  |  |
| **1** | Yes |
| **2** | No **[Go to A21]** |

|  | | **Hypertension** | **Diabetes** | **Hypercho-lesterolemia** | **Heart Disease** | **Stroke** | **Renal Failure** | **Asthma** | **COPD** |
| --- | --- | --- | --- | --- | --- | --- | --- | --- | --- |
| A20 How often is your blood pressure  monitored at home? | |  |  |  |  |  |  |  |  |
| **1** | Daily |
| **2** | More than once a week |
| **3** | Once a week |
| **4** | Every 2 weeks |
| **5** | Once a month |
| **6** | Every 2 months |
| **7** | Routinely once every few months |
| **8** | As and when necessary |
| A21 Why is your blood pressure not  monitored at home?  **(List all that apply)** | | **[Go to Sect.C]** |  |  |  |  |  |  |  |
| **1** | Don’t know how to take |
| **2** | No time to take |
| **3** | Feel better |
| **4** | Don’t bother to |
| **5** | Go somewhere else to monitor |
| **6** | Expensive to buy BP monitor |
| **7** | Was not informed by healthcare professional to do so |
| **8** | Others **[Specify in column]** |
| A22 Do you monitor your blood  glucose at home? | |  |  |  |  |  |  |  |  |
| **1** | Yes |
| **2** | No **[Go to A24]** |
| A23 How often is your blood glucose  monitored at home? | |  | **[Go to A25]** |  |  |  |  |  |  |
| **1** | Daily |
| **2** | More than once a week |
| **3** | Once a week |
| **4** | Every 2 weeks |
| **5** | Once a month |
| **6** | Every 2 months |
| **7** | Routinely once every few months |
| **8** | As and when necessary |
| A24 Why is your blood glucose not  monitored at home?  **(List all that apply)** | |  |  |  |  |  |  |  |  |
| **1** | Don’t know how to take |
| **2** | No time to take |
| **3** | Feel better |
| **4** | Don’t bother to |
| **5** | Go somewhere else to monitor |
| **6** | Expensive to buy glucometer / glucose strips |
| **7** | Was not informed by healthcare professional to do so |
| **8** | Others **[Specify in column]** |
| A25 Are you on insulin? | |  |  |  |  |  |  |  |  |
| **1** | Yes |
| **2** | No **[End]** |
|  | | **Hypertension** | **Diabetes** | **Hypercho-lesterolemia** | **Heart Disease** | **Stroke** | **Renal Failure** | **Asthma** | **COPD** |
| A26 Who administers your insulin? | |  |  |  |  |  |  |  |  |
| **1** | Self |
| **2** | Family member |
| **3** | Caregiver (non-family member) |
| **4** | Others **[Specify in column]** |
| A27 What problems do you have in  administering insulin?  **(List all that apply)** | |  | **[Go to Sect.C]** |  |  |  |  |  |  |
| **1** | None |
| **2** | Injecting too much / too little |
| **3** | Forgetting to inject |
| **4** | Finding someone who can inject |
| **5** | Running out of insulin |
| **6** | Unable to see the measurement |
| **7** | Others **[Specify in column]** |

| **B. Health Seeking Behaviour** |
| --- |

B1 Do you have a medical condition which requires regular attention (treatment / monitoring) by a healthcare professional?

1. Yes **[Specify condition(s)]**: ____________________
2. No **[Go to B3]**

B2 Where do you usually go to seek treatment for your medical condition? **[Go to B4]**

**(Tick all that apply)**

1. Private GP (Family Clinic)
2. Polyclinic
3. SOC – Restructured Hospital **[Specify name]**: _______________
4. SOC – Private Hospital
5. Traditional medicine practitioner
6. Emergency Department
7. Self-medicate
8. Did not seek treatment at all
9. Others: ____________________

B3 Where are you likely to go if you have a medical condition?

1. Private GP (Family Clinic)
2. Polyclinic
3. SOC – Restructured Hospital **[Specify name]**: _______________
4. SOC – Private Hospital
5. Traditional medicine practitioner
6. Emergency Department
7. Self-medicate
8. Would not seek treatment at all
9. Others: ____________________

B4 How many times have you sought treatment at the following for any medical condition in the

past 12 months?

times

times

times

times

times

times

times

- Private GP (Family Clinic)
- Polyclinic
- SOC – Restructured Hospital **[Specify name]**: _______________
- SOC – Private Hospital
- Traditional medicine practitioner **[To answer B6 also]**
- Emergency Department
- Others : _______________________

B5 What are the reasons for choosing the most frequently visited healthcare provider (B4):

____________________? **(Tick all that apply)**

1. Cheaper
2. More accessible
3. Condition can be better managed
4. Doctor-related factor
5. Service quality
6. More advanced technology
7. Reputation
8. Efficiency
9. Loyalty / familiarity
10. Recommendation
11. Others: ____________________

B6 Why did you visit a traditional medicine practitioner instead of a Western doctor?

**(Tick all that apply)**

1. Sought treatment from BOTH traditional medicine practitioner

and Western doctor

1. Cheaper
2. More accessible
3. Condition can be better managed
4. Practitioner-related factor
5. Loyalty / familiarity
6. Western medical treatment not effective
7. Want a second opinion
8. For body modulation
9. Others: ____________________

B7 What kind(s) of assistance do you need most when you seek treatment / in your daily life? **(Tick all that apply)**

1. None
2. Social activities
3. Emotional support
4. Transportation
5. Financial assistance
6. Health programs (screening / talk)
7. Caregiver (feeding / transferring / toileting /

dressing & undressing / bathing)

1. Infrastructural support (eg. hand rails / ramps)
2. Others: ____________________

B8 How do you usually pay for your outpatient fees (consultation / medication / lab tests) to the healthcare provider? **(Tick all that apply)**

1. Out of own pocket
2. Company pays for it
3. Medisave
4. Insurance
5. Medifund
6. Paid by children / relatives
7. Other government scheme: ____________________
8. Other non-government scheme: ____________________
9. Don’t know

B9 How many times in the past 12 months were you admitted into a hospital?

1. Never **[Go to Section C]**
2. 1 time; Name of hospital: ____________________
3. 2 times; Name of hospital(s): ____________________
4. ≥ 3 times; Name of hospital(s): ____________________

B10 What were the reasons for your admission into hospital? **(List)**

__________________________________________________________________________

__________________________________________________________________________

| **C. Lifestyle and Other Risk Factors** |
| --- |

C1 How often do you exercise?

1. Regularly – any form of sports for ≥ 20 minutes per occasion, ≥ 3 days a week **[Go to C3]**
2. Occasionally – any form of sports for ≥ 20 minutes per occasion, < 3 days a week **[Go to C3]**
3. Not at all but perform some form of physical activity such as stretching and bending
4. Not at all

C2 What are your reason(s) for not exercising? **(Tick all that apply)**

1. No time due to work / family commitments
2. No companion to exercise with
3. Too lazy
4. Too tired because of work / family commitments
5. Too old
6. Poor health
7. Doctor advised not to exercise
8. Have enough physical activity at work / home
9. Lack of facilities
10. Weather is too hot / humid
11. Don’t know any exercises
12. Others: ____________________

C3 Do you walk for at least 10 minutes continuously to travel from one place to another?

1. Yes
2. No **[Go to C5]**

C4 In a usual week, on how many days do you walk for at least 10 minutes at a time from one place to another?

days

C5 Have you ever smoked cigarettes?

1. Yes
2. No **[Go to Section D]**

C6 How frequent do you smoke?

1. Daily (no. of cigarettes per day): __________
2. Occasionally
3. Have stopped smoking completely **[Go to C10]**

C7 What are your reasons for smoking? **(Tick all that apply)**

1. To feel relaxed / relieve stress / help cope with problems
2. To help me concentrate
3. Would feel unbearable if I do not smoke
4. Smoking is enjoyable
5. Boredom
6. To feel confident / grown up / important
7. To be like my family members / relatives
8. To model film / TV stars
9. To impress my boyfriend / girlfriend / friends / colleagues
10. To entertain clients / friends
11. Others: ____________________

C8 What is stopping you from quitting smoking? **(Tick all that apply)**

1. Nothing – I believe I can stop anytime I want to
2. Difficult to resist the urge
3. No urgent need to stop
4. Others: ____________________

C9 Which of the following best describes your plan about smoking? **[Go to Section D]**

1. I plan to quit smoking within the next month
2. I plan to quit smoking within the next 6 months
3. I plan to quit smoking within the next 12 months
4. I plan to quit smoking within the next 5 years
5. I plan to quit smoking sometime in the future
6. I do not plan to quit smoking at all but plan to cut down on the number of cigarettes smoked
7. I do not plan to quit smoking at all and do not plan to cut down on the number of cigarettes smoked

C10 What are the reasons why you stopped smoking completely? **(Tick all that apply)**

1. Advised to stop smoking by my doctor
2. Learnt about the harmful effects of smoking
3. Health reasons / experienced the ill effects of smoking
4. Concerned about the health of those around me (passive smoking)
5. Cigarettes have become too expensive
6. Smoking is a waste of money
7. Pressure / advice to stop from family / friends / colleagues
8. No particular reason / decided to give up voluntarily
9. Others: ____________________

| **D. Functional Assessment** |
| --- |

INTERVIEWERS: Please tick in the boxes accordingly.

D1 Do you have any progressive/permanent functional decline in the past 12 months?

1. Yes **[Specify duration]**: __________
2. No

D2 Are you able to perform the following activities?

| **Basic ADL** | **Current** | | |
| --- | --- | --- | --- |
|  | **Independent** | **Assistance** | **Dependent** |
| Feeding |  |  |  |
| Transferring |  |  |  |
| Toileting |  |  |  |
| Dressing / Undressing |  |  |  |
| Bathing |  |  |  |

| **Instrumental ADL** | **Current** | | |
| --- | --- | --- | --- |
|  | **Independent** | **Assistance** | **Dependent** |
| Using telephone |  |  |  |
| Travelling (public transport) |  |  |  |
| Shop for groceries |  |  |  |
| Preparing meals |  |  |  |
| Do housework |  |  |  |
| Taking medicine |  |  |  |
| Managing money |  |  |  |

D3 Ambulatory Status

1. Independent – Community ambulant
2. Requiring assistance
3. Non-ambulant – Chair bound / Bed bound **[Go to D5]**

D4 Mobility Aid

1. None
2. Walking stick
3. Quad stick
4. Walking frame
5. Others: ____________________

D5 In the past 12 months, have you fallen to the ground or fallen and hit something like a chair or the stairs?

1. Yes **[Specify date of last fall]**: ____________________
2. No

D6 In the past 12 months, have you stumbled or lost your balance, but did not actually fall to the ground?

1. Yes
2. No

D7 In the past 12 months, have you ever lost control of urine and got wet?

1. Yes
2. No **[Go to D10]**

D8 Have you lost control of urine on at least 6 separate days?

1. Yes
2. No

D9 How bothersome is this symptom?

1. Not bothersome
2. Slightly bothersome
3. Bothersome
4. Very bothersome

D10 Has there been any progressive forgetfulness? *(To be corroborated with family members if available)*

1. Yes **[Specify duration]**: __________
2. No

D11 Do you often feel sad or depressed?

1. Yes
2. No

D12 Without trying, have you lost 5kg or more in weight in the last 6 months?

1. Yes **[how much]**: __________ kg

Patient’s weight: __________ kg

Duration: __________

1. No
2. Not sure

D13 Do you often cough / choke on food / liquid?

1. Yes **[how often]**: __________
2. No

D14 Do you have difficulty doing any of your daily activities because of your eyesight, even while wearing glasses?

1. Yes
2. No

D15 Do you have difficulty hearing?

1. Yes
2. No

D16 Are you using any hearing aid?

1. Yes
2. No

| **E. Participation in Health Programs** |
| --- |

INTERVIEWERS: Please complete all questions for each program before moving on to the next

|  | | | **Screening Program** | **Talks / Seminars / Public Forum** | **Workshops** |
| --- | --- | --- | --- | --- | --- |
| E1 Would you be willing to participate in a  health program for chronic diseases? | | |  |  |  |
| **1** | Yes **[Go to E3]** | |
| **2** | No | |
| E2 Why would you not be willing to participate  in the health program? **[Go to Section F]**  **(List all that apply)** | | |  |  |  |
| **1** | | No time |
| **2** | | Not willing to pay |
| **3** | | Not interested |
| **4** | | No one to bring me for it |
| **5** | | Unwilling to travel |
| **6** | | Anxiety about the procedure in the screening program |
| **7** | | Others: ____________________ |
| E3 How much will you be willing to pay for the  health program? | | |  |  |  |
| **1** | | $0 (free of charge) |
| **2** | | $1 to < $5 |
| **3** | | $5 to < $10 |
| **4** | | $10 to < $15 |
| **5** | | $15 to < $20 |
| **6** | | > $20 |
| E4 Where would you want the  health program to be conducted?  **(List all that apply)** | | |  |  |  |
| **1** | | Community centres |
| **2** | | HDB void deck |
| **3** | | Mobile bus |
| **4** | | Residential Committee (RC) centres |
| **5** | | Atriums of residential shopping malls |
| **6** | | Polyclinics |
| **7** | | Others **[Specify in column]** |

| **F. Overall rating** |
| --- |

F1 Rate current state of health **(Tick in the appropriate box)**

**1 2 3 4 5 6 7 8 9 10**

**Worst Best**

**possible possible**

| **G. Demographics** |
| --- |

G1 Name: ____________________________________________________________________

G2 NRIC No:

D D M M Y Y Y Y

G3 Date of Birth (dd-mm-yyyy):

G4 Ethnic Group

1. Chinese
2. Malay
3. Indian
4. Others

G5 Highest education level attained

1. No formal qualifications
2. Primary (PSLE)
3. Secondary (‘O’ / ‘N’ Levels)
4. Junior college (‘A Levels)
5. Diploma / Other professional qualification
6. University & Higher

G6 Main language medium

1. English
2. Mandarin
3. Malay
4. Tamil
5. Dialect: ____________________

G7 Currently staying with **(Tick all that apply)**

1. No one
2. Spouse
3. Children
4. Relatives
5. Friends
6. Tenant
7. Maid
8. Others: ____________________

G8 Main work status over past 12 months

1. Working full-time **[occupation]**: ____________________
2. Working part-time **[occupation]**: ____________________
3. Student
4. National Service Man
5. Housewife
6. Retiree
7. Unemployed **[previous occupation]**: ____________________
8. Unemployed (due to disability or other medical conditions)

G9 Your average monthly income* received from each of the following

**(Specify according to options provided)**

Self: ( ) **1** None

Spouse: ( ) **2** < $300

Children: ( ) **3** $300 to < $500

Relatives: ( ) **4** $500 to < $800

Friends: ( ) **5** $800 to < $1,000

Other sources (eg. Charitable organizations): **6** $1,000 to < $2,000

Organisation 1: ____________________ ( ) **7** $2,000 to < $3,000

Organisation 2: ____________________ ( ) **8** $3,000 to < $4,000

**9** ≥ $4,000

** refers to salary / annuities / rental / investment returns / monetary allowances received*

G10 Currently on any social support programme

1. Yes

Name of organization: ______________________________

Nature of the program: ______________________________

1. No

Name of interviewer : ________________________

Signature : ________________________

Date : ________________________
